# Association of long-term 5α-reductase inhibitor use with survival in men with renal cell carcinoma: a nationwide population-based cohort study

**DOI:** 10.3389/fphar.2026.1811121

**Published:** 2026-05-22

**Authors:** Sieun Lee, Jooyoung Lee, Kyungchan Min, Jong Hyun Tae, Chung Un Lee, Joongwon Choi, Jung Hoon Kim, Yong Seong Lee, Tuan Thanh Nguyen, Se Young Choi

**Affiliations:** 1 Department of Applied Statistics, Chung-Ang University, Seoul, Republic of Korea; 2 Department of Urology, Chung-Ang University Hospital, Chung-Ang University College of Medicine, Seoul, Republic of Korea; 3 Chung-Ang University Gwangmyeong Hospital, Chung-Ang University College of Medicine, Gwangmyeong, Gyeonggi-do, Republic of Korea; 4 Department of Urology, Cho Ray Hospital, University of Medicine and Pharmacy at Ho Chi Minh City, Ho Chi Minh City, Vietnam; 5 MADI Inc., Seoul, Republic of Korea

**Keywords:** 5-alpha reductase inhibitors, androgen receptor, drug repurposing, overall survival, real-world evidence, renal cell carcinoma

## Abstract

**Background:**

The incidence and mortality of renal cell carcinoma (RCC) exhibit marked sex-based differences, suggesting a potential role for androgen signaling in disease progression. Although 5α-reductase inhibitors (5-ARIs) are widely prescribed for benign prostatic conditions, their potential association with survival in patients with RCC remains poorly understood. We evaluated the association between long-term use of 5-ARIs and overall survival in men with RCC.

**Methods:**

This population-based retrospective cohort study used data from the Korean National Health Insurance Service database (2007–2020). Men aged ≥40 years with newly diagnosed RCC were included. Long-term 5-ARI use was defined as cumulative prescription of finasteride or dutasteride for ≥365 days prior to diagnosis. Propensity score matching (1:3 ratio) was employed to balance baseline characteristics, including age, year of diagnosis, income, comorbidity burden, and hypertension. The primary outcome was all-cause mortality, analyzed using Cox proportional hazards regression models. Subgroup analyses were stratified by treatment modality and Charlson comorbidity index (CCI).

**Results:**

Among 31,927 eligible men, 1,803 5-ARI users were matched with 5,409 non-users (mean age 71.2 years). Long-term 5-ARI use was associated with significantly lower all-cause mortality (hazard ratio [HR]: 0.88; 95% CI: 0.79–0.94; p < 0.001). Although no significant associations were observed in patients undergoing surgery (HR: 0.94; 95% CI: 0.79–1.07) or systemic therapy (HR: 1.15; 95% CI: 0.82–1.55), a distinct survival benefit was evident in the no-surgery/no-systemic therapy group (HR: 0.82; 95% CI: 0.73–0.92; p < 0.001). Within this untreated subgroup, the benefit was most pronounced in patients with low comorbidity (CCI 0–1: HR: 0.78; 95% CI: 0.65–0.91) compared with those with high comorbidity (CCI ≥2: HR: 0.89; 95% CI: 0.76–1.03).

**Conclusion:**

Long-term 5-ARI use is associated with improved overall survival in men with RCC, particularly among those with low comorbidity who are managed without active oncological treatment. These findings suggest that androgen modulation may influence RCC progression and support the investigation of 5-ARIs as potential adjunctive agents in selected clinical settings.

## Introduction

Over the past few decades, the incidence of renal cell carcinoma (RCC) has increased, and RCC is now the fourteenth most common malignancy worldwide (Bray et al., 2024). This increase is attributed to population aging, lifestyle factors, and the widespread use of advanced imaging, which have led to more frequent detection of small renal masses (Abdelaziz et al., 2025). Although many of these tumors are incidentally discovered and clinically localized at diagnosis and substantial progress has been made in surgical and systemic therapy, RCC remains a major contributor to global cancer mortality (Bray et al., 2024).

The incidence and mortality of RCC exhibit marked sex-based differences, with men being nearly twice as likely as women to develop the disease and die from RCC (Bray et al., 2024). This disparity suggests a potential role for sex hormones and androgen signaling in the carcinogenesis and progression of RCC (Ladurner et al., 2024). Experimental studies have shown that activation of the androgen receptor (AR), which is expressed in both normal and malignant renal tissues, promotes RCC cell proliferation, epithelial–mesenchymal transition, and angiogenesis (You et al., 2021). Conversely, suppression of AR signaling by enzalutamide or abiraterone *in vivo* can inhibit tumor growth (Lee et al., 2017). However, clinical evidence regarding the role of sex hormones in RCC remains limited and controversial. Exogenous estrogen exposure through oral contraceptives or pregnancy may alter RCC risk in women (Kabat et al., 2007), whereas hormone-related studies in men, who have a substantially higher incidence of RCC, remain scarce.

Dihydrotestosterone (DHT), a metabolite of testosterone, is a critical component of the androgen axis and binds to the AR with higher affinity than testosterone, thereby enhancing downstream signaling (Askew et al., 2007). Of note, 5α-reductase inhibitors (5-ARIs), such as finasteride and dutasteride, are widely prescribed to treat benign prostatic hyperplasia and androgenetic alopecia by blocking this conversion (Shin et al., 2025). Preclinical studies and *in vitro* models have provided a theoretical basis for investigating the effect of 5-ARIs on RCC. By suppressing DHT synthesis, 5-ARI use may attenuate pro-tumorigenic androgen signaling within RCC, thereby influencing tumor biology and improving outcomes (Pak et al., 2019). This hormonal mechanism provides a biologically plausible hypothesis for hypothesizing a beneficial effect of long-term 5-ARI use in male patients with RCC.

Clinical data on the association between 5-ARI use and RCC prognosis are scarce and inconclusive, and prior studies have been limited by small sample sizes or lack of longitudinal exposure data (Lipponen et al., 2025). Therefore, we conducted a large-scale, population-based retrospective cohort study using data from the Korean National Health Insurance Service (NHIS) database to determine whether long-term 5-ARI use is associated with improved overall survival among male patients with RCC.

## Methods

### Data source

This population-based retrospective cohort study utilized data from the NHIS database of South Korea, which covers approximately 99% of the Korean population and includes information on inpatient and outpatient diagnoses, prescription medications, and hospitalizations. Diagnoses were identified using the International Classification of Diseases, 10th Revision (ICD-10) codes. A customized dataset was constructed from the NHIS database to include patients with RCC diagnosed between 2007 and 2020.

This study was approved by the institutional review board at the Chung-Ang University Hospital (IRB No. 2307-027-19481). Given its retrospective design and the use of de-identified administrative data, the requirement of obtaining informed consent was waived. The study adhered to the principles of the Declaration of Helsinki.

### Study population

The study population consisted of male patients aged ≥40 years who were newly diagnosed with RCC (ICD-10 code C64) between 2007 and 2020. Exclusion criteria were as follows: a history of any cancer diagnosis prior to RCC diagnosis (n = 22,971), and prior 5α-reductase inhibitor (5-ARI) prescription with a cumulative duration <365 days before RCC diagnosis (n = 4,250).

### Exposure

The primary exposure was cumulative 5-ARI use for at least 365 days prior to RCC diagnosis. The 5-ARIs included finasteride and dutasteride, identified using prescription codes in the NHIS database. Cumulative exposure was calculated as the sum of prescription days from the first 5-ARI prescriptions to the date of RCC diagnosis. Patients were classified as 5-ARI users (cumulative use ≥365 days) or non-users (no prescription history). Patients with <365 days of cumulative use were also excluded.

### Outcomes and covariates

The primary outcome was all-cause mortality, ascertained from the NHIS death registry. The index date was defined as the date of RCC diagnosis for the overall cohort. Patients were followed from the index date until death from any cause or the end of the study period (31 December 2020), whichever came first.

Secondary outcomes included treatment-related complications and resource utilization: (1) hospital readmission within 90 days of surgery, (2) length of post-surgery hospital stay (analyzed both as a continuous variable and as a binary variable ≤ 7 days vs. > 7 days), (3) transfusion requirement, and (4) transfusion volume.

Surgical treatment was defined as the initial surgical intervention, including open or laparoscopic nephrectomy and robot-assisted nephrectomy. Open and laparoscopic nephrectomies were identified using procedure codes in the NHIS database. Robot-assisted nephrectomy is generally not reimbursed by the NHIS; therefore, we identified it indirectly as cases with a general anesthesia claim recorded concurrent with hospitalization for RCC, without any reimbursed nephrectomy procedure codes. The corresponding disease and treatment codes are listed in Supplementary Table S1.

Demographic variables included age at diagnosis (<55, 55–64, 65–74, and ≥75 years) and year of diagnosis (2007–2011, 2012–2016, and 2017–2020). Socioeconomic status was categorized into low, middle, and high based on insurance premium levels. Comorbidity burden was assessed using the Charlson comorbidity index (CCI) and categorized as 0–1, 2–3, or ≥4. Hypertension was included as an additional covariate because it is not accounted for in CCI calculation.

### Statistical analysis

Baseline characteristics were compared between the 5-ARI users and non-users using chi-square tests for categorical variables and Student’s *t*-tests for continuous variables. Propensity scores were estimated using a logistic regression model, with the following categorical variables: age at diagnosis, year of diagnosis, income level, CCI, and hypertension. One-to-three nearest-neighbor matching without replacement was performed.

Covariate balance after matching was assessed using standardized mean differences (SMDs) for all baseline covariates, with absolute SMD values <0.1 considered acceptable balance. Overall survival was analyzed using Kaplan–Meier methods, and survival curves were compared using the log-rank test. Cox proportional hazards regression models were used to estimate hazard ratios (HRs) and 95% confidence intervals (CIs) for the association between 5-ARI use and mortality.

Secondary outcomes were analyzed using linear regression models for continuous variables (length of hospital stay and transfusion volume) and logistic regression models for binary variables (readmission, hospital stay ≤7 days, and transfusion requirement). Results were reported as mean differences with 95% CIs for continuous variables and odds ratios (ORs) with 95% CIs for binary variables.

All CIs for the matched cohort analyses were calculated using 500 bootstrap replicates to account for the matching procedure. Statistical significance was set at *p* < 0.05 (two-sided). Analyses were performed using R version 4.0.3 (R Foundation for Statistical Computing, Vienna, Austria) and SAS Enterprise Guide version 7.1 (SAS Institute Inc., Cary, NC).

### Subgroup analyses

Subgroup analyses were performed for the surgery, systemic therapy, and no-surgery/no-systemic-therapy groups. The surgery group included patients who underwent partial or radical nephrectomy, and the systemic therapy group included patients who received systemic therapy. The no-surgery/no-systemic-therapy group included patients who received neither surgical nor systemic therapy. The index date was defined as the date of nephrectomy for the surgery subgroup and the date of first systemic therapy administration for the systemic therapy subgroup.

Due to heterogeneity in the no-surgery/no-systemic-therapy group, further stratification was performed by comorbidity status: low CCI (0–1) and high CCI (≥2).

## Results

### Baseline characteristics

**FIGURE 1:**
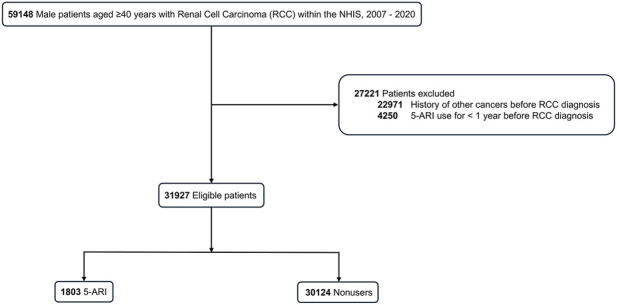
Patient selection flow chart. Among 59,148 patients aged ≥40 years diagnosed with renal cell carcinoma (RCC) in the National Health Insurance Service (NHIS) database between 2007 and 2020, 31,927 met the eligibility criteria and were included in the analysis: 1,803 5-α reductase inhibitor (5-ARI) users and 30,124 non-users.

Among the 59,148 patients aged ≥40 years diagnosed with RCC, 31,927 met the eligibility criteria and were included in the study: 1,803 (5.6%) 5-ARI users and 30,124 (94.4%) non-users (Figure 1).

Before propensity score matching, substantial differences were observed between groups, with large SMDs indicating imbalance. The 5-ARI user group had a higher proportion of older patients, greater comorbidity burden, and more prevalent hypertension than the non-user group.

After 1:3 propensity score matching, 1,803 patients in the 5-ARI user group were matched with 5,409 non-users. Baseline characteristics after matching were well balanced, with all SMDs <0.1 (Table 1).

**TABLE 1:** Baseline characteristics of enrolled patients with RCC before and after propensity score matching.

Characteristic	Patient no. (%)					
	Before propensity score matching			After propensity score matching		
	5-ARI user group (N = 1,803)	Non-user group (N = 30,124)	SMD	5-ARI user group (N = 1,803)	Non-user group (N = 5,409)	SMD
Age (years)	​	​	1.34	​	​	0.06
0 (<55)	65 (3.6)	11,988 (39.8)	​	65 (3.6)	195 (3.6)	​
1 (55–65)	291 (16.1)	9,417 (31.3)	​	291 (16.1)	873 (16.1)	​
2 (65–75)	703 (39.0)	5,929 (19.7)	​	703 (39.0)	2,259 (41.8)	​
3 (≥75)	744 (41.3)	2,790 (9.3)	​	744 (41.3)	2,082 (38.5)	​
Diagnosis year	​	​	0.31	​	​	0.03
0 (2007–2011)	326 (18.1)	9,167 (30.4)	​	326 (18.1)	1,005 (18.6)	​
1 (2012–2016)	653 (36.2)	10,539 (35.0)	​	653 (36.2)	2,000 (37.0)	​
2 (2017–2020)	824 (45.7)	10,418 (34.6)	​	824 (45.7)	2,404 (44.4)	​
Income	​	​	0.15	​	​	0.04
Low	541 (30.0)	7,903 (26.2)	​	541 (30.0)	1,524 (28.2)	​
Middle	420 (23.3)	8,997 (29.9)	​	420 (23.3)	1,283 (23.7)	​
High	842 (46.7)	13,224 (43.9)	​	842 (46.7)	2,602 (48.1)	​
Charlson comorbidity index	​	​	0.74	​	​	0.02
0 (0–1)	238 (13.2)	12,332 (40.9)	​	238 (13.2)	714 (13.2)	​
1 (2–3)	644 (35.7)	10,571 (35.1)	​	644 (35.7)	1,975 (36.5)	​
2 (≥4)	921 (51.1)	7,221 (24.0)	​	921 (51.1)	2,720 (50.3)	​
Hypertension	1,375 (76.3)	13,960 (46.3)	0.32	428 (23.7)	4,092 (75.7)	0.01

SMD, standardized mean difference.

### Overall survival

#### Entire matched cohort

**FIGURE 2:**
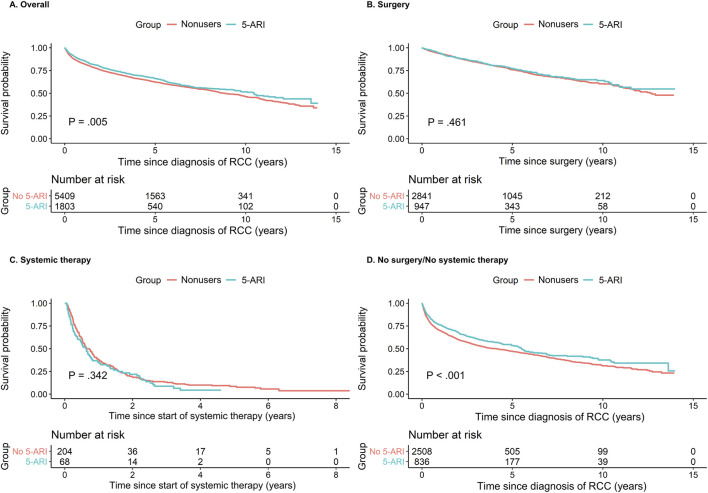
Overall survival by 5-ARI use in patients with RCC. Kaplan–Meier curves for overall survival according to 5-ARI use in **(A)** the overall cohort, **(B)** the surgery subgroup, **(C)** the systemic therapy subgroup, and **(D)** the no-surgery/no-systemic therapy subgroup after propensity score matching. Survival time was measured from the date of RCC diagnosis **(A,D)**, surgery **(B)**, or systemic therapy initiation **(C)**. *p*-values were obtained using the log-rank test. Propensity score matching was performed, adjusted for age, year of diagnosis, income, Charlson comorbidity index, and hypertension. The no-surgery/no-systemic therapy subgroup includes patients who did not receive definitive oncologic treatment during the study period.

**FIGURE 3:**
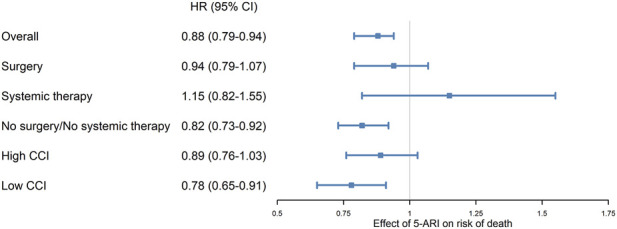
Association of 5α-reductase inhibitor (5-ARI) use with overall survival in patients with RCC: overall and subgroup analyses. Forest plot showing hazard ratios (HRs) and 95% confidence intervals (CIs) for overall survival comparing patients with 5-ARI use versus non-users after propensity score matching. Subgroup analyses using treatment modality and Charlson comorbidity index (CCI) in the no-surgery/no-systemic-therapy group are shown. HRs were estimated using Cox proportional hazard models in the matched cohort.

During a mean follow-up of 3.7 years, 2,536 deaths occurred in the matched cohort: 578 (32.1%) in the 5-ARI user group and 1,958 (36.2%) in the non-user group. The mortality rate was lower in the 5-ARI user group than in the non-user group (86.5 vs. 98.3 per 1,000 person-years). Kaplan–Meier curves demonstrated clear divergence between groups (Figure 2A; *p* = 0.005). In Cox regression analysis, 5-ARI use was associated with a reduced risk of death (Figure 3; HR: 0.88; 95% CI: 0.79–0.94; p < 0.001).

#### Surgery subgroup

Among patients who underwent nephrectomy, 947 patients in the 5-ARI user group and 19,087 in the non-user group were identified before matching. After matching, 947 patients in the 5-ARI user group were matched with 2,841 in the non-user group (Supplementary Table S2). Kaplan–Meier analysis showed no significant difference in survival between the groups (Figure 2B; *p* = 0.461). Cox regression analysis also showed no significant difference (Figure 3; HR: 0.94; 95% CI: 0.79–1.07; *p* = 0.456).

Secondary outcomes showed no significant differences (Table 2). Readmission occurred more frequently in the 5-ARI user group; however, the difference was not significant (OR: 1.17; 95% CI: 0.98–1.34; *p* = 0.054). The proportion of patients with hospital stay ≤7 days was comparable between the two groups (OR: 1.05; 95% CI: 0.87–1.25; *p* = 0.554). Length of hospital stay and transfusion outcomes did not significantly differ (Table 2).

**TABLE 2:** Effects of 5α-reductase inhibitor use on surgical and systemic therapy outcomes.

Outcome	Effect size (95% CI)	*p*-value
Surgery subgroup		
Readmission	1.17 (0.98–1.34)	0.05
Length of hospital stay (≤7 days)	1.05 (0.87–1.25)	0.55
Length of hospital stay	0.13 (−0.27–0.58)	0.66
Transfusion	0.90 (0.76–1.10)	0.22
Transfusion (mL)	−14.98 (−32.72–14.13)	0.23
Death	0.94 (0.79–1.07)	0.46
Systemic therapy subgroup		
Transfusion	1.16 (0.31–2.61)	0.71
Transfusion (mL)	201.90 (−33.24–298.81)	>0.99
Death	1.15 (0.82–1.55)	0.44

CI, confidence interval.

#### Systemic therapy subgroup

Among patients who received systemic therapy, 68 in the 5-ARI user group and 1,346 in the non-user group were identified before matching. After matching, 68 patients in the 5-ARI user group were matched with 204 in the non-user group (Supplementary Table S2). Kaplan–Meier analysis showed no difference in survival between groups (Figure 2C; *p* = 0.342). Cox regression analysis also showed no significant association (Figure 3; HR: 1.15; 95% CI: 0.82–1.55; p = 0.44). Secondary outcomes did not differ significantly between groups (Table 2).

#### No-surgery/no-systemic therapy subgroup

Among patients who received neither surgical nor systemic therapy, 836 patients in the 5-ARI user group and 10,730 in the non-user group were identified before matching. After matching, 836 patients in the 5-ARI user group were matched with 2,508 in the non-user group (Supplementary Table S2). Kaplan–Meier survival analysis showed longer overall survival in the 5-ARI user group than in the non-user group. Cox regression analysis indicated a reduced risk of death for the 5-ARI user group than for the non-user group (Figure 3; HR: 0.82; 95% CI: 0.73–0.92; p < 0.001).

#### Comorbidity stratification for the no-surgery/no-systemic therapy group

Among patients with low CCI (0–1) in the no-surgery/no-systemic therapy group, 354 patients in the 5-ARI user group and 7,881 in the non-user group were identified before matching. After matching, 354 patients in the 5-ARI user group were matched with 1,062 patients in the non-user group. Furthermore, 5-ARI use was associated with a survival benefit (Figure 3; HR: 0.78; 95% CI: 0.65–0.91; p = 0.002).

Among patients with high CCI (≥2) in the no-surgery/no-systemic therapy group, 482 patients in the 5-ARI user group and 2,849 in the non-user group were identified before matching. After matching, 482 patients in the 5-ARI user group were matched with 1,446 in the non-user group. No significant association between 5-ARI use and mortality was observed (Figure 3; HR: 0.89; 95% CI: 0.76–1.03; p = 0.12).

## Discussion

In this large, population-based cohort study using NHIS data, long-term use of 5-ARIs was associated with improved overall survival among male patients with RCC. The survival benefit was most pronounced in patients who did not undergo surgical or systemic therapy, whereas no significant association was observed among those who did. These findings suggest that suppression of androgen activity through long-term 5-ARI exposure may be associated with improved outcomes in specific subgroups of patients with RCC, although the underlying biological mechanisms were not directly evaluated in this study.

To our knowledge, this is the first large-scale, real-world study to demonstrate a potential survival benefit of 5-ARI use in male patients with RCC. Our findings contrast with those of some previous epidemiological reports. For instance, Kabra et al. (2018), using self-reported medication data from the Prostate, Lung, Colon, and Ovarian Cancer Screening Trial, reported no association between 5-ARI use and RCC incidence. Doherty et al. (2023) reported that, in men with benign prostatic hyperplasia, 5-ARI use was not associated with an increased risk of kidney cancer compared with alpha-blocker use. Lipponen et al. (2025), analyzing data from the National Finnish Cancer Registry, observed that pre-diagnostic 5-ARI use was linked to an increased risk of RCC-related death, with the risk increasing with longer duration or greater cumulative 5-ARI use. This adverse association was observed in subgroups of patients who did not undergo surgery and in those with clear cell RCC histology, a pattern that stands in direct opposition to the survival benefit observed in our no-surgery/no-systemic-therapy subgroup. This discrepancy may stem from racial differences, variations in healthcare systems, lifestyle factors, and differences in competing mortality risks across populations (Kotecha et al., 2024; Collaborators, 2025a; Collaborators, 2025b). More recently, Cheng et al. (2025) suggested a possible immunological mechanism through which androgen modulation could enhance therapeutic response in metastatic RCC. The authors reported that prior 5-ARI exposure was associated with favorable changes in the tumor immune microenvironment, including AR-related CD8^+^ T-cell activity, ultimately leading to improved responsiveness to immune checkpoint inhibitors. Taken together, these conflicting epidemiological findings and complex mechanistic data underscore that the role of 5-ARIs in RCC prognosis is intricate and context-dependent, varying across treatment settings. Therefore, further research should focus on clarifying the therapeutic role of 5-ARIs by rigorously assessing their efficacy within specific, detailed clinical contexts and patient subgroups.

To provide a broader epidemiological context, it is informative to consider the prevalence of 5-ARI use in the general Korean population. A recent nationwide study on men aged over 40 who underwent PSA screening reported that 39.4% had been prescribed 5-ARIs (Pyun et al., 2024). In our RCC cohort, long-term 5-ARI users (defined as ≥365 days of cumulative use) accounted for 5.6% of the patients. The difference between the general exposure rate and the proportion in our study likely reflects our stringent definition of long-term exposure, which excludes transient or short-term users to better capture the sustained pharmacologic impact of androgen suppression. In addition, exposure to 5-ARIs was defined based on prescription records, and actual medication adherence or refill compliance could not be directly assessed. Although we required a minimum cumulative exposure of 365 days to identify long-term users, some degree of misclassification of true drug exposure may remain. This approach ensures that the observed survival benefit is associated with meaningful clinical exposure rather than sporadic medication use.

The subgroup analyses provide additional insights into the potential mechanisms underlying the observed association between 5-ARI use and improved survival. The absence of a significant survival difference among patients who underwent nephrectomy or received systemic therapy suggests that the effects of 5-ARI may be relatively modest when the disease is surgically removed or when potent oncological treatments dominate the prognosis. Conversely, the no-surgery/no-systemic-therapy subgroup showed a clear positive association with 5-ARI use. This finding should be interpreted with caution as it may reflect differences in underlying disease stage, treatment eligibility, or clinical management, rather than a direct pharmacologic effect of 5-ARIs alone. In this group, including those on active surveillance or unsuitable for aggressive treatment, the anticancer effect of 5-ARIs may translate into a measurable survival benefit by stabilizing disease progression. However, due to the limitations of claims-based data, we were unable to distinguish between active surveillance and watchful waiting or to assess specific follow-up protocols and monitoring strategies. Furthermore, stratifying the no-surgery/no-systemic therapy group by CCI provided critical insight into the effect of modification. Although the low CCI group (0–1) showed the strongest survival benefit, the association was attenuated and non-significant in the high CCI group (≥2). This pattern may reflect the influence of competing risks of death, although alternative explanations, including residual confounding and differences in clinical characteristics, cannot be excluded. Patients with a low CCI have better overall health status, with mortality risk predominantly driven by RCC progression, allowing the beneficial anticancer effect of 5-ARIs to be clearly expressed in the survival curve. Because 5-ARI users and non-users were balanced for comorbidity burden through propensity score matching, the survival advantage observed within the low-CCI subgroup suggests a drug-specific effect rather than a simple reflection of lower comorbidity. Patients with a low CCI in the no-surgery/no-systemic therapy group likely represent a population undergoing active surveillance for small renal masses. In this context, 5-ARIs may act as chemopreventive or stabilizing agents, delaying tumor growth in early-stage disease, whereas their effect is negligible in advanced stages that require systemic therapy. In contrast, patients with a high CCI have a significantly elevated risk of death from non-cancer causes, including severe cardiovascular events or other chronic diseases. In this group, the beneficial effect of 5-ARIs is likely masked by competing mortality risks (Kutikov et al., 2012). This detailed subgroup analysis strongly suggests that 5-ARIs may have a selectively beneficial role in patients with RCC having relatively good general health but are not receiving definitive surgical or systemic therapy.

Our study possesses several notable strengths. First, we utilized data from the large, population-based Korean NHIS database, which covers nearly the entire Korean population, minimizing selection bias and ensuring high generalizability to the real-world population of men with RCC.

Second, using objective administrative claims data rather than self-reported surveys, we ensured more accurate ascertainment of diagnoses, longitudinal prescription history, and all-cause mortality. Third, we implemented a rigorous methodological approach, employing propensity score matching (1:3) using nearest-neighbor matching without replacement to effectively balance baseline characteristics between the 5-ARI users and non-users, thereby reducing confounding bias.

Despite the large sample size and propensity score matching, our study has some limitations inherent to its retrospective, observational design. First, as this was an observational study, we could only demonstrate an association between long-term 5-ARI use and overall survival and could not establish causality. In addition, the primary outcome was all-cause mortality rather than cancer-specific mortality. As administrative data do not reliably capture the cause of death, the observed associations may not fully reflect cancer-specific survival differences and may be influenced by non-cancer-related factors, including comorbidity burden and competing risks of death. Second, using data from the NHIS claims database limits access to crucial clinical data. We could not incorporate essential oncological factors, such as tumor stage, histological subtype, and Fuhrman grade, which are key prognostic determinants in RCC. The absence of these variables introduces the possibility of residual confounding that cannot be fully addressed in our analysis. In particular, the no-surgery/no-systemic therapy subgroup may include patients with heterogeneous clinical indications, introducing potential selection bias that cannot be fully accounted for in claims-based analyses. In addition, the interpretation of secondary outcomes related to postoperative complications and resource utilization is limited by the lack of detailed clinical information, such as tumor stage and surgical complexity, and therefore should be interpreted with caution. Third, we could not adjust for important lifestyle and biological variables that might act as unmeasured confounders, including smoking status, body mass index, physical activity, and detailed information regarding the indication for 5-ARI prescription. Finally, although we required cumulative use of at least 1 year to define long-term exposure, there remains a possibility of confounding by indication, in which 5-ARI use might simply be a surrogate marker for proactive health-seeking behavior or higher socioeconomic status. We acknowledge that the observed overall hazard ratio (HR 0.88) represents a modest reduction in mortality risk. Although statistically significant in this large-scale cohort, its clinical significance should be interpreted with caution as it may not justify a change in management for all patients with RCC. Future studies incorporating longitudinal imaging data and tumor-specific outcomes are needed to clarify the underlying mechanisms.

## Conclusion

This large-scale, real-world cohort study provides population-level evidence of a possible association between long-term use of 5-ARIs and improved overall survival among male patients with RCC. The survival benefit was particularly evident in patients who did not undergo surgical or systemic therapy and in those with a lower comorbidity burden, suggesting that hormonal modulation through 5-ARI exposure may influence disease biology or overall health status in selected populations. Although the present findings do not establish causality, they highlight a previously under-recognized link between androgen signaling and RCC outcomes. These results warrant further investigation through prospective cohort studies and mechanistic research incorporating molecular and immunological markers to elucidate how androgen suppression affects tumor progression and therapeutic responsiveness. Ultimately, understanding the interplay between androgen regulation and RCC pathophysiology may facilitate the development of individualized, hormone-based adjunctive strategies for RCC management.

## Data Availability

The datasets presented in this article are not readily available because the data that support the findings of this study were provided by the National Health Insurance Service (NHIS) of South Korea. Restrictions apply to the availability of these data, which were used under license for the current study and are not publicly available. Data are, however, available from the authors upon reasonable request and with permission of the NHIS. Requests to access the datasets should be directed to the corresponding author, SC (urosyc@cau.ac.kr). Please note that the data were obtained from the National Health Insurance Service (NHIS) of South Korea, and any secondary use of these data requires formal approval from the NHIS. Researchers may also apply for data access directly through the NHIS Data Sharing Service website (https://nhiss.nhis.or.kr/).
