# Supplementary material for: Association of long-term 5α-reductase inhibitor use with survival in men with renal cell carcinoma: a nationwide population-based cohort study
Source: Front Pharmacol. 2026 May 22;17:1811121. doi: 10.3389/fphar.2026.1811121 (PMC13236679; doi:10.3389/fphar.2026.1811121)
Supplement: Supplementary file 1 [file Table1.docx]

**Supplementary Table**

**e Table 1. ICD-10 and drug codes for defining the study population, interventions, outcomes, and comorbidities**

|  | Code |
| --- | --- |
| Renal cell carcinoma (RCC) | C64 |
| Partial nephrectomy | R3290 |
| Radical nephrectomy | R3273 |
| Charlson comorbidity index (CCI) |  |
| Myocardial infarction | I21, I22, I252 |
| Congestive heart failure | I43, I50, I099, I110, I130, I132, I255, I420, I425, I426-I429, P290 |
| Peripheral vascular disease | I70, I71, I731, I738, I739, I771, I790, I792, K551, K558, K559, Z958, Z959 |
| Cerebrovascular disease | G45, G46, I60-I69, H340 |
| Dementia | F00-F03, G30, F051, G311 |
| Chronic pulmonary disease | J40-J47, J60-J67, I278, I279, J684, J701, J703 |
| Rheumatologic disease | M05, M06, M32-M34, M315, M351, M353, M360 |
| Peptic ulcer disease | K25-K28 |
| Mild liver disease | B18, K73, K74, K700-K703, K709, K713-K715, K717, K760, K762-K764, K768, K769, Z944 |
| Diabetes without chronic complications | E100, E101, E106, E108-E111, E116, E118-E121, E126, E128-E131, E136, E138-E141, E146, E148, E149 |
| Diabetes with chronic complications | E102-E105, E107, E112-E115, E117, E122-E125, E127, E132-E135, E137, E142-E145, E147 |
| Hemiplegia or paraplegia | G81, G82, G041, G114, G801, G802, G830-G834, G839 |
| Renal disease | N18, N19, N052-N057, N250, I120, I131, N032-N037, Z490-Z492, Z940, Z992 |
| Any malignancy, including leukemia and lymphoma | C00-C26, C30-C34, C37-C41, C43, C45-C58, C60-C76, C81-C85, C88, C90-C97 |
| Moderate or severe liver disease | K704, K711, K721, K729, K765-K767, I850, I859, I864, I982 |
| Metastatic solid tumour | C77-C80 |
| AIDS/HIV | B20-B24 |
| Hypertension | I10-I13, I15 |
| Transfusion | X1001, X1002, X2021, X2022 |
| Dutasteride | 458801ACS |
| Finasteride | 159001ATB |
| Sunitinib malate | 487701ACH, 487702ACH, 487703ACH |
| Pazopanib hydrochloride | 611801ATB, 611802ATB |
| Temsirolimus | 568201BIJ, 568230BIJ |
| Ipilimumab | 633330BIJ, 633331BIJ |
| Nivolumab | 638401BIJ, 638402BIJ |

**eTable 2. Baseline characteristics of renal cell carcinoma (RCC) patients with no-surgery/no-systemic therapy before and after propensity score matching**

| Covariate | Patients, No. (%) | | | | | |
| --- | --- | --- | --- | --- | --- | --- |
|  | Before propensity score matching | | | After propensity score matching | | |
|  | 5-ARI user group  (N=836) | Non-user group  (N=10,730) | SMD | 5-ARI user group  (N=836) | Non-user group  (N=2,508) | SMD |
| Age |  |  | 1.29 |  |  | 0.08 |
| 0 (< 55) | 16 (1.9) | 3,647 (34) |  | 16 (1.9) | 48 (1.9) |  |
| 1 (55–64) | 97 (11.6) | 3,027 (28.2) |  | 97 (11.6) | 291 (11.6) |  |
| 2 (65–74) | 258 (30.9) | 2,335 (21.8) |  | 258 (30.9) | 863 (34.4) |  |
| 3 (> 75) | 465 (55.6) | 1,721 (16) |  | 465 (55.6) | 1,306 (52.1) |  |
| Diagnosis year |  |  | 0.32 |  |  | 0.06 |
| 0 (2007–2011) | 151 (18.1) | 3,351 (31.2) |  | 151 (18.1) | 475 (18.9) |  |
| 1 (2012–2016) | 262 (31.3) | 3,099 (28.9) |  | 262 (31.3) | 839 (33.5) |  |
| 2 (2017–2020) | 423 (50.6) | 4,280 (39.9) |  | 423 (50.6) | 1,194 (47.6) |  |
| Income |  |  | 0.16 |  |  | 0.04 |
| Low | 261 (31.2) | 3,047 (28.4) |  | 261 (31.2) | 740 (29.5) |  |
| Middle | 183 (21.9) | 3,100 (28.9) |  | 183 (21.9) | 585 (23.3) |  |
| High | 392 (46.9) | 4,583 (42.7) |  | 392 (46.9) | 1,183 (47.2) |  |
| Charlson's comorbidity index |  |  | 0.79 |  |  | 0.05 |
| 0 (0–1) | 92 (11) | 4,107 (38.3) |  | 92 (11) | 277 (11) |  |
| 1 (2–3) | 261 (31.3) | 3,748 (35) |  | 261 (31.3) | 842 (33.6) |  |
| 2 (> 4) | 483 (57.7) | 2,875 (26.8) |  | 483 (57.7) | 1,389 (55.4) |  |
| Hypertension | 672 (80.4) | 5,087 (47.4) | 0.73 | 672 (80.4) | 1,967 (78.4) | 0.05 |

*Abbreviation: SMD, standardized mean difference.*
